# Exploring the reciprocal relationship between activities of daily living disability and depressive symptoms among middle-aged and older Chinese people: a four-wave, cross-lagged model

**DOI:** 10.1186/s12889-023-16100-0

**Published:** 2023-06-20

**Authors:** Jiayi Wang, Nansheng Luo, Yu Sun, Ru Bai, Xueying Li, Libing Liu, Hui Wu, Li Liu

**Affiliations:** Department of Social Medicine, School of Health Management, China Medical University, No. 77 Puhe Road, Shenyang North New Area, Shenyang, 110122 Liaoning China

**Keywords:** Depressive symptoms, Activities of daily living, Reciprocal relationship, Cross-lagged model, Middle-aged and older people, Longitudinal study

## Abstract

**Background:**

Early studies have shown a relationship between activities of daily living (ADL) disability and depressive symptoms in older people. However, discussions on the direction of this relationship are insufficient. The study’s objective was to assess the reciprocal relationship between ADL disability and depressive symptoms among middle-aged and older Chinese people.

**Method:**

Data was collected in four waves of a nationwide survey, the China Health and Retirement Longitudinal Study (CHARLS), which was carried out in 2011, 2013, 2015, and 2018. In total, this study included 4,124 participants aged ≥ 45 years at baseline. A summing score of the eleven items for basic activities of daily living (BADL) and instrumental activities of daily living (IADL) was calculated to indicate the degree of ADL disability. The 10-item Centre for Epidemiological Studies Depression Scale (CESD-10) was adopted to measure depressive symptoms. The reciprocal relationship between ADL disability and depressive symptoms was tested by cross-lagged models.

**Result:**

At baseline, 911 (22.1%) participants were classified as having difficulties with ADL, and the prevalence of depressive symptoms was 34.4% (1,418). Among middle-aged and older people in China, there was a significant reciprocal and longitudinal relationship between ADL disability and depressive symptoms. People who had difficulty with ADL faced a higher risk of depressive symptoms, and those who suffered from depressive symptoms were accompanied by an increase in ADL disability in the following years. The subgroup analysis on age also showed that ADL disability was reciprocally and longitudinally related to depressive symptoms. However, only women showed similar results in the subgroup analysis on gender.

**Conclusion:**

This study shows that ADL disability is bi-directionally related to depressive symptoms in middle-aged and older Chinese people over time. The results suggest we should identify ADL disability and bad psychological conditions in time to prevent subsequent mutual damage among middle-aged and older Chinese people, a vulnerable group rising in the future.

**Supplementary Information:**

The online version contains supplementary material available at 10.1186/s12889-023-16100-0.

## Introduction

Depression is a common mental disorder that seriously limits physiological and psychosocial functions and undermines the quality of human life [1]. Among the causes of disability-adjusted life years between 1990 and 2019, depressive disorder is responsible for the most increase. As one of the ten leading causes of increasing burden, depressive disorder is common across young, middle-aged, and older adults [2]. Globally, it is estimated that 5% of adults suffer from depression [3]. Depression is predominant among older people, with the same scale (Geriatric Depression Scale, GDS-15) finding a prevalence of depressive symptoms among middle-aged and older people of 9.2% in Sweden [4], 7.55% in Portugal [5] and 17.4% in China [6]. Older people have a higher incidence of depressive symptoms [7]. Further, among Chinese people over 45 years old, the prevalence of depressive symptoms is 26.67% and 38.37% for men and women, respectively [8]. Middle-aged and older Chinese people show more severe depressive symptoms than the US, UK, and Mexico [9]. As the population ages, the increasing incidence of depressive symptoms in older people has already been a major public health issue in China. It is vital to target risk causes to prevent depressive symptoms. Previous research has identified a number of risk factors for depressive symptoms, including females, separated/divorced/widowed marital status, loneliness, poor cognition, mobility and ADL disability [10, 11].

Activities of daily living (ADL) can be interpreted as basic activities that people must do daily to survive and adapt to their environment. ADL covers activities such as movement, self-care, communication, and housework [12]. ADL generally comprises basic activities of daily living (BADL) and instrumental activities of daily living (IADL). BADL focuses on self-care skills, including bathing, dressing, and going to the toilet [13]. IADL includes more complicated behaviours such as cooking, money management, and shopping [14]. The top four reasons associated with ADL disability were age, pain, taking more medicines, and depression in community-dwelling older people [15]. A cross-country comparison showed that among older people in six middle-income countries, old age is one of the most common causes of ADL-related disabilities [16]. Having difficulty with ADL is common among older people [11]. Keeping the ability to perform ADL is fundamental to independent living for older people, which is essential to reducing the potential care burdens on families and the state.

Previous studies showed ADL disability and depressive symptoms can significantly interact with each other [17, 18]. Beck [19] also provides a sound theoretical basis for exploring the bidirectional relationship between depressive symptoms and ADL disability. Beck proposed a cognitive-theoretical model of depression. In this model, he used dysfunctional attitudes as a susceptibility factor. Individuals with depressive tendencies develop dysfunctional attitudes when exposed to stressful events (e.g., I seek help as a sign of weakness) [20]. Then individuals may develop corresponding negative beliefs that interact with other cognitive, affective, motivational, and behavioural systems [21]. It eventually leads to clinical somatic disorders (e.g., ADL disability), reduced motivation, and depressed mood. These symptoms, in turn, lead to increased negative cognitions, forming a vicious cycle with an upward trend [22]. Therefore, clarifying the bidirectional causal relationship between depressive symptoms and ADL disability is necessary to identify important factors that break this vicious circle in the future.

Previous research has found a correlation between ADL disability and depressive symptoms. A cross-sectional study of 5,863 older adults found an association between depressive symptoms and ADL disability in Chinese older adults [11]. Kim et al. found a significant correlation between ADL ability and depressive symptoms through a cross-sectional analysis [23]. However, the study only explored the point-in-time correlation and did not clarify the causal relationship. Subsequently, scholars became aware of the need for longitudinal studies. Fan et al. found people with severe ADL disability had a subsequent increased risk of depressive symptoms based on a longitudinal group-based trajectory model [24]. Using a longitudinal mediation model, Peng et al. clarified that the onset of depressive symptoms increases the risk of subsequent ADL disability in older adults [25]. While explaining cause and effect, the above longitudinal studies are confined to a one-way relationship. Chen et al. [26] and Yang et al. [27] proved a bidirectional relationship between ADL disability and depressive symptoms through longitudinal studies. However, studies were limited to Taiwan’s older adults and geriatric arthritis patients, and the national replication is weak. The knowledge of the relationship between ADL disability and depressive symptoms is still limited. Therefore, it is essential to identify the bidirectional causal relationship between ADL disability and depressive symptoms in middle-aged and older people using a large and representative sample.

In the present study, we used cross-lagged model to examine a reciprocal causal relationship exists between ADL disability and depressive symptoms among middle-aged and older people in China during 2011 to 2018. Unlike traditional cohort studies, the cross-lagged model focuses on the interaction between variables while taking into account the effect of the variable's pre-test state on the post-test, ensuring the continuity and stability of the analysis [28].

We hypothesized that ADL disability at baseline would predict the occurrence of subsequent depressive symptoms. In the same way, depressive symptoms at baseline would predict the following changes in ADL disability.

## Method

### Participants

A longitudinal study with a follow-up survey was conducted. The study is based on four waves of longitudinal data from the China Health and Retirement Longitudinal Study (CHARLS) conducted by Peking University from 2011 to 2018. CHARLS conducted a multi-stage sample of 150 counties and 450 communities in 28 provinces across China. Samples of adults aged 45 and above were selected for face-to-face interviews and questionnaires with individuals and their families. It conducts follow-up visits every two years to collect information on the participants' demographic characteristics and social, economic, and health functioning status [29].

Based on the study’s purpose, the following inclusion criteria were set up: (1) age ≥ 45 years old in the 2011 survey. (2) participation in all 2011–2018 four follow-up surveys. (3) The database contains four rounds of complete responses from participants on demographic backgrounds such as gender, education, marital status, and health status and functional information such as sleep, smoking, and alcohol consumption. (4) no missing values for measures of ADL (DB010–DB020) and depressive symptoms (DC009–DC018). Li Liu and Jiayi Wang extracted the data. After obtaining the CHARLS data, we combined the data from the four waves and filtered the participants who took part in all four waves of the survey. Next, the study variables were selected from the data, while data cleaning and data coding were then performed to obtain the final data. There were 17,708 individuals were recruited in baseline (2011) survey. Firstly, 11,982 participants who participated in all four waves of the survey were enrolled. A total of 4,539 participants with complete depression scale were elected in all four waves. Then, 4,527 participants remained after deleting those with missing values on the ADL scale in the four waves. Participants with no missing values on the demographic variables and older than 45 years at baseline were selected. Finally, 4,124 participants were included in the study. We compared the baseline characteristics of the 4,124 participants in the final analysis with the 7,858 participants who took part in four waves of the survey but were excluded due to missing data. Results showed no significant differences between the two groups on locality, nap after lunch, tobacco use, alcohol use, and chronic disease (Additional Table 1).

### Measurements

#### ADL disability

The measurement of ADL ability was divided into BADL and IADL. BADL includes the ability to eat, bathe, dress, get in and out of bed, go to the toilet, and defecate. IADL includes the ability to do housework, shop, cooking, take medicine, and manage personal property. Participants needed to respond, “Do you have difficulty with completing some daily acts, for physical, mental, emotional or memory reasons?” Four answers include (1) do it without any difficulty; (2) still can do it despite difficulty; (3) have difficulty and need others’ help; and (4) unable to do it. According to each item of BADL/IADL, when respondents reported no difficulty with the activity, 0 was recorded. If respondents reported difficulty with or could not do the activity, 1 was recorded. The total scores of ADL disability were determined by summing scores of the eleven items, ranging from 0 to 11. Cronbach’s alpha coefficients were 0.820, 0.790, 0.805, and 0.829, respectively.

#### Depressive symptoms

At four time points, the depressive symptoms of 4,124 participants were measured by the 10-item Centre for Epidemiological Studies Depression Scale (CESD-10). Eight negative items and two positive items were included in the questionnaire of CESD-10. Participants were asked to rate "In the past week, how often did you feel?" with scores ranging from 0 (< 1 day) to 3 (5–7 days). The scores on responses for two positive items were reversed. Total score ranges from 0 to 30, with higher scores predicting higher levels of depressive symptoms. In this study, depressive symptoms were measured by scoring 10 and higher on CESD-10. CSED-10 has good validity and reliability among older Chinese people [30]. In the four waves of the survey, Cronbach's alpha coefficients were 0.805, 0.761, 0.792, and 0.802, respectively.

#### Covariates

Age, sex, the highest level of education (divided into four groups: not finish primary school and lower; graduate from elementary school; graduate from middle school; high school and above), hours of actual sleep at night [< 6 h; 6 h– (include 6 h); 7 h– (include 7 h); > 8 h], marital status (have a spouse or not), locality (rural or urban), nap after lunch (yes or no), tobacco use (yes or no), alcohol use (yes or no), chronic disease (yes or no), and social activity (yes or no). Chronic disease was defined as a respondent being diagnosed with one of the 14 common chronic diseases, such as hypertension, dyslipidaemia, diabetes, cancer, heart attacks, and emotional problems. Social activity was measured based on the question, “Have you done any of these activities in the last month?” We considered the respondent has social activity as long as he or she participated in one of the 11 common social activities.

### Statistical analysis

The continuous variables were described by the mean and standard deviation (SD), and the categorical variables were expressed as frequency and percentage. We performed regression analyses for ADL disability and depressive symptoms, adjusting for all covariates and creating regression equations at baseline and three follow-up visits (2011, 2013, 2015, and 2018), respectively. Then, we saved residuals and standardized them with *Z*-transformation (mean = 0; standard deviation = 1). Analyses were conducted by IBM SPSS statistical software version 21.0. We used an auto-regressive cross-lagged path model to test changes in participants' ADL disability and depressive symptoms in 2011, 2013, 2015, and 2018. Amos 25.0 was used to establish the cross-lagged model and estimated path coefficients. The indexes of a good fitting model were: Chi-square degree of freedom ratio (χ^2^/df) values less than 5, Comparative Fit Index (CFI), as well as Tucker Lewis Index (TLI), values greater than 0.95, Root Mean Square Error of Approximation (RMSEA) values less than 0.07, and Standardized Root Mean-square Residual (SRMR) less than 0.08 [26]. The Fisher *Z* test was used to test the difference in the directional relationship between ADL disability and depressive symptoms as derived from the *Z* values, which shows more robust evidence of their temporary relationship. Two-tailed *P* < 0.05 was considered statistically significant. Subgroup analyses were also conducted on sex differences and age differences (baseline age < 60; ≥ 60) in participants.

## Result

Table 1 illustrates the characteristics of the participants. A total of 4,124 participants were included in the four waves of the survey. At baseline, participants were aged between 45 and 89 years (Mean = 57.57 years, SD = 7.89), 50.6% of whom were female, 39.5% of participants had not finished primary school and lower, and nearly half of the participants (43.5%) maintained 7-8 h of sleep per night. Over fifty percent of the participants lived in rural areas, took naps, did not smoke or drink, and had a chronic illness and social activity.

**Table 1:** Characteristics of participants at baseline (*N* = 4,124)

Variables	Mean (SD)	%
Age	57.57 (7.89)	
Gender		
Male		49.4
Female		50.6
Locality		
Rural		64.0
Urban		36.0
Education		
Not finish primary school and lower		39.5
Graduate from elementary school		24.6
Graduate from middle school		22.7
High school education and above		13.2
Marital status		
Have a spouse		91.6
Not have a spouse		8.4
Hours of actual sleep at night		
< 6 h		27.4
6 h– (include 6 h)		21.1
7 h– (include 7 h)		43.5
> 8 h		8.0
Nap after lunch		
Yes		50.8
No		49.2
Tobacco use		
Yes		32.9
No		67.1
Alcohol use		
Yes		39.4
No		60.6
Chronic disease		
Yes		68.1
No		31.9
Social activity		
Yes		54.2
No		45.8

Table 2 showed the descriptive statistics of study variables in four waves. There were undulations in both average scores of ADL disability and depressive symptoms. The average scores of ADL disability ranged from 0.54±1.41 at baseline to 0.45±1.21 in 2013, 0.69±1.54 in 2015, and 0.84±1.76 in 2018. The average score of CESD-10 decreased from 8.00±6.19 in 2011 to 7.82±5.75 and then increased to 8.00±6.30 in 2015 and 8.91±6.69 in 2018.

**Table 2:** Mean values and percentages for ADL disability and depressive symptoms at each wave (*N* = 4,124)

Health Status	Year	Mean (SD)	%
ADL disability (0–11)	2011	0.54 (1.41)	22.1
	2013	0.45 (1.21)	20.4
	2015	0.69 (1.54)	27.7
	2018	0.84 (1.76)	30.2
Depressive symptoms (0–30)	2011	8.00 (6.19)	34.4
	2013	7.82 (5.75)	31.7
	2015	8.00 (6.30)	34.1
	2018	8.91 (6.69)	39.6

CESD-10 scores ≥ 10 was regarded as having depressive symptoms; *ADL* disability scores ≥ 1 was regarded as having difficulties in ADL

**Fig. 1:**
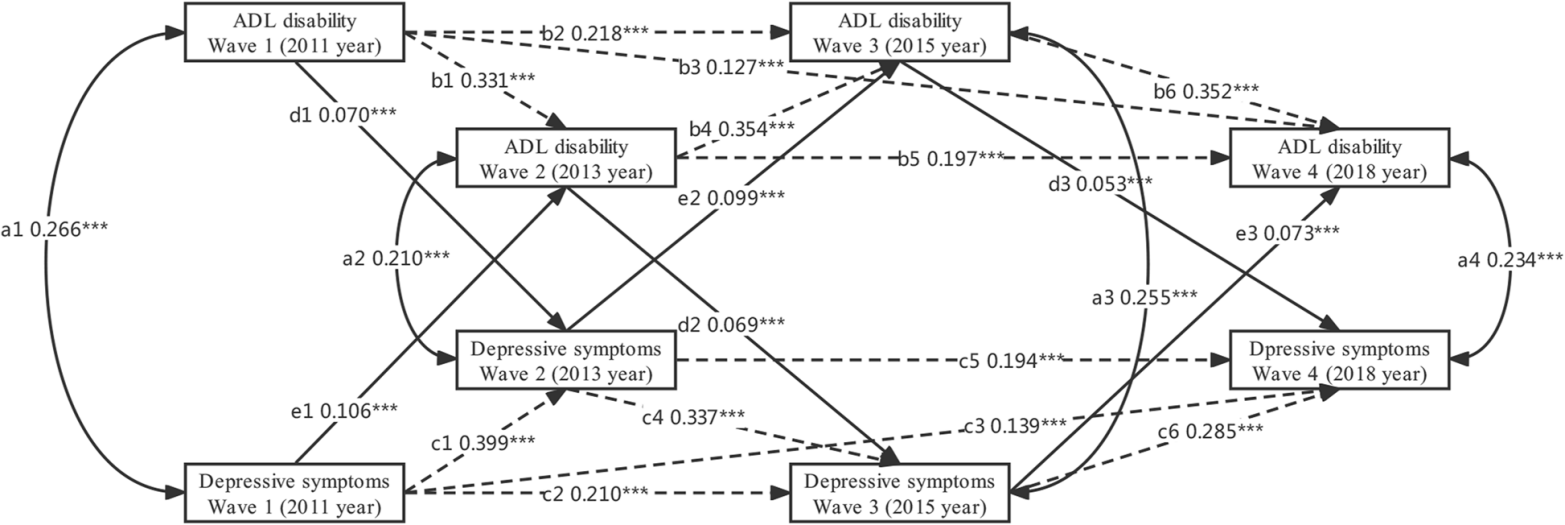
A cross-lagged and autoregression model for estimation between ADL disability and depressive symptoms over four waves during 2011 and 2018. Dotted lines with one head represent auto-regressive paths (paths b1-b6 and c1-c6). Solid lines with one head represent cross-lag paths (paths d1, d2, d3 and e1, e2, e3). Concurrent associations among variables during the same wave are represented by solid lines with two heads (paths a1, a2, a3, and a4). Covariates included age, gender, locality, education, hours of actual sleep at night, marital status, nap after lunch, tobacco use, alcohol use, chronic disease, and social activity. Continuous variable: ADL disability and depressive symptoms. The path coefficients shown are standardized. ***P < 0.001. ADL: activities of daily living

Figure 1 presents the model fit index of the autoregressive cross-lagged model for ADL disability and depressive symptoms. The result showed for data: χ^2^ = 28.622, df = 6, χ^2^/df = 4.770, CFI = 0.986, TLI = 0.997, RMSEA = 0.030, and SRMR = 0.014. The model meets the fit criteria and has a good fit. The paths d1 (β = 0.070, SE = 0.015, *P* < 0.001), d2 (β = 0.069, SE = 0.014, *P* < 0.001), d3 (β = 0.053, SE = 0.014, *P* < 0.001) from ADL disability to depressive symptoms between 2011 and 2018 were statistically significant. Likewise, there were positive paths e1 (β = 0.106, SE = 0.015, *P* < 0.001), e2 (β = 0.099, SE = 0.014, *P* < 0.001), e3 (β = 0.073, SE = 0.014, *P* < 0.001) from depressive symptoms to ADL disability. The Fisher *Z* test indicated that the path coefficients from ADL disability to follow-up depressive symptoms and from depressive symptoms to follow-up ADL disability were not statistically different.

**Fig. 2:**
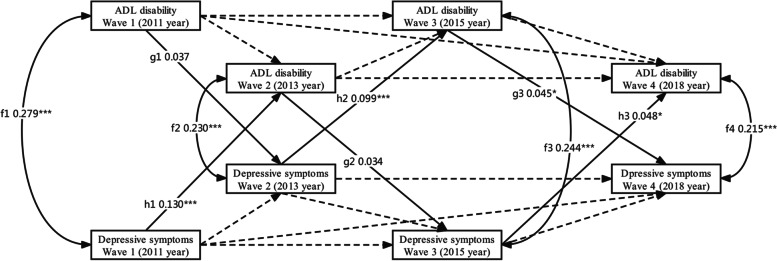
A cross-lagged and autoregression model for estimation between ADL disability and depressive symptoms over four waves during 2011 and 2018 in men. Dotted lines with one head represent auto-regressive paths. Solid lines with one head represent cross-lag paths. Concurrent associations among variables during the same wave are represented by solid lines with two heads. To simplify the presentation, this model only presents cross-lag paths (paths g1, g2, g3 and h1, h2, h3) and concurrent paths between variables at the same survey (paths f1, f2, f3, and f4). Covariates included age, gender, locality, education, hours of sleep at night, marital status, nap after lunch, tobacco use, alcohol use, chronic disease, and social activity. Continuous variable: ADL disability and depressive symptoms. The path coefficients shown are standardized. *, *P* < 0.05; **, *P* < 0.01; ***, *P* < 0.001. ADL: activities of daily living

**Fig. 3:**
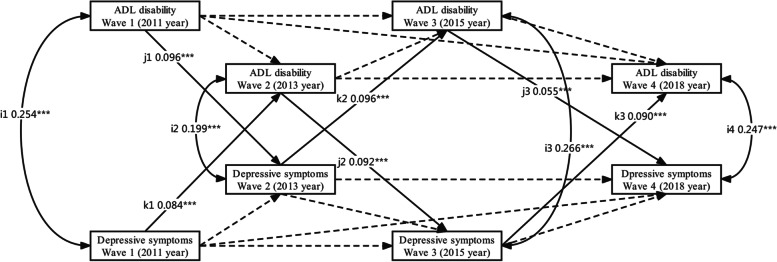
A cross-lagged and autoregression model for estimation between ADL disability and depressive symptoms over four waves during 2011 and 2018 in women. Dotted lines with one head represent auto-regressive paths; Solid lines with one head represent cross-lag paths; Concurrent associations among variables during the same wave are represented by solid lines with two heads. To simplify the presentation, this model only presents cross-lag paths (paths j1, j2, j3 and k1, k2, k3) and concurrent paths between variables at the same survey (paths i1, i2, i3, and i4). Covariates are age, gender, locality, hours of sleep at night, education, marital status, nap after lunch, tobacco use, alcohol use, chronic disease, and social activities. Continuous variable: ADL disability and depressive symptoms. The path coefficients shown are standardized. ***P < 0.001. ADL: activities of daily living

In Figs. 2 and 3, a subgroup analysis was carried out on sex. The results showed that paths from ADL disability to depressive symptoms from 2011 to 2015 are not statistically significant (*P* > 0.05) in men (χ^2^ = 22.072, df = 6, χ^2^/df = 3.679, CFI = 0.996, TLI = 0.981, RMSEA = 0.036, SRMR = 0.016). Fisher *Z* test indicated that between 2015 and 2018, the path coefficients from ADL disability to follow-up depressive symptoms (g3) and from depressive symptoms to follow-up ADL disability (h3) in men were not statistically different. In women, all cross-lagged paths are statistically significant (χ^2^ = 12.879, df = *6*, χ^2^/df = 2.147, CFI = 0.998, TLI = 0.991, RMSEA = 0.023, SRMR = 0.012). Also, the Fisher *Z* test indicated that the path coefficients from ADL disability to follow-up depressive symptoms and from depressive symptoms to follow-up ADL disability in women were not statistically different.

The subgroup analysis in age is similar to the main analysis presented: (under 60 years old: χ^2^/df = 5.034, CFI = 0.995, TLI = 0.977, RMSEA = 0.039, SRMR = 0.015; 60 years old and above: χ^2^/df = 1.689, CFI = 0.998, TLI = 0.993, RMSEA = 0.021, SRMR = 0.012). All cross-lagged paths are statistically significant (*P* < 0.001).

## Discussion

Using the representative sample from CHARLS, we found that difficulties with ADL and depressive symptoms are common in middle-aged and older Chinese people [31]. Between 2011 and 2018, ADL disability and depressive symptoms scores fluctuated. We suspect that China revised the *Elderly rights law* in 2012, which clearly stated that “Family members should care for the mental needs of the elderly and shall not neglect them.” and “Government encourages organizations and individuals to provide services such as spiritual comfort and psychological counselling to older people at home [32].” This law gave more protection to the mental health of older people than before. It may be related to reduced ADL difficulties and depressive symptoms among participants in 2013. Further, we found a positive, reciprocal, time-varying association between ADL disability and depressive symptoms. More severe depressive symptoms predicted higher amounts of difficulties in ADL longitudinally as time went by. In the reverse direction, higher levels of ADL disability longitudinally predicted an increased risk of depressive symptoms.

In theory, two directions of this relationship are reasonable according to middle-aged and older people. Older people’s ADL abilities are reduced due to the deterioration of physical functions [33]. Having more difficulties with ADL increases the risk of depression by limiting older people's physical activity and social networks [34]. Over time, ADL disability can increase the incidence of depressive symptoms. Studies found that people with ADL difficulties, such as dressing, bathing, and toilet use, are at higher risk of depressive symptoms [35]. Besides, those with more difficulties in ADL and who need ADL assistance from others had higher depressive symptoms [36]. The reasons may be physical limitations in self-care activities, such as difficulties getting out of bed and going to the toilet. Older people with difficulties in ADL may not receive enough social support, a protective factor for depression [37–39]. ADL disability may act as a psychological stressor because of loss of independence and ability to care for oneself. It reduces energy, motivation, effort, and ability to interact with others resulting in depressive symptoms [7, 30].

Similarly, researchers have also found that depressive symptoms are a necessary predictor of difficulties with ADL [40]. Previous studies showed that people who felt depressed were more likely to report having difficulty with ADL ability [41]. By various potential mechanisms, depressive symptoms can influence ADL disability. For instance, depressive symptoms can act as a stressor, causing and aggravating inflammatory processes, thereby increasing the risk for ADL disability subsequently. Somatic symptoms of depression, such as fatigue and aches, may also cause a decline in physical functioning in older people, undermining ADL ability [42]. Besides, depressive symptoms can accelerate the development of ADL disability through social and psychological mechanisms [12, 15]. Individuals who suffer from depression are often less likely to have long-term adherence to medical recommendations [27], usually lose hope in life, and lack motivation and vigour for social [13, 43] and physical activities [14, 27], accordingly undermining ADL ability.

In the subgroup analysis, ADL disability does not predict the following depressive symptoms in men between 2011–2015. Possible reasons include women being more susceptible to the harmful impacts of role overload and violence, and hormonal fluctuations can also increase the risk of depression in women during perimenopause [44]. Another explanation could be that when experiencing negative emotions, women's response style tends to ruminate and think about possible causes, which increases their susceptibility to depression. In contrast, men tried to distract themselves and lift their moods by seeking social support or engaging in activities with friends [45]. However, with age, men are more likely to have more depressive symptoms than women when facing difficulties, possibly due to a decline in health that limits participation in social activities [46]. Therefore, the prediction of ADL disability on depressive symptoms will gradually appear as older men grow.

The presence of depressive symptoms in the middle-aged and older population is closely associated with “Wish to Die” [47] and significantly increases the risk of death [48]. Identifying potential depressive symptoms in older people is vital for early prevention and treatment. We should pay more attention to older people with ADL disabilities to avoid the onset of their depressive symptoms. Similarly, ADL disability is also an independent risk factor for five-year mortality in older people [49]. Depressive symptoms can be a strong predictor for the prevention of ADL disability in older adults. The results of this study suggest that older adults with depressive symptoms and ADL disability are both at high risk and need more attention. This study only examined the bivariate relationship between ADL disability and depressive symptoms. Future work is needed to identify the influences of this relationship to break this vicious circle once and for all.

We should mention a few limitations of this study. First, when considering chronic disease as a covariate, CHARLS counted data related to 14 major categories of chronic diseases. To avoid too many variables, we used the presence or absence of chronic diseases rather than the specific type of chronic disease suffered. However, some disease types have a more significant impact on mobility, for example, joint disease. We will refine the model in the future by incorporating different classifications of chronic diseases. Second, this study excludes the missing data, which would have resulted in the loss of some samples and affected the analysis results. Third, we used four waves of data from the CHARLS database, which used self-reporting and may have partial information bias.

## Conclusion

In summary, the result of the study suggests that changes in ADL disability propel changes in depressive symptom in middle-aged and older people and vice versa. The interaction between the ADL disability and depressive symptom may trigger a vicious circle. In practice, early interventions targeting depressive symptoms or ADL disability can be beneficial in improving the health of middle-aged and older people. Therefore, we should clarify how ADL disability and depressive symptoms interact with each other to prevent the development of a vicious cycle among middle-aged and older Chinese people, a vulnerable group rising in the future.

## Supplementary Information

**Additional file 1: Additional Fig. 1.** An estimated autoregressive cross-lagged model of ADL disability and depressive symptoms over four waves between 2011 and 2018 in people with baseline age <60. **Additional Fig. 2.** An estimated autoregressive cross-lagged model of ADL disability and depressive symptoms over four waves between 2011 and 2018 in people with baseline age ≥60. **Additional Tab. 1.** Baseline characteristics of 4,124 study participants included in final analyses and 7,858 excluded due to data missing in the CHARLS.

## Data Availability

The datasets that support the findings of this study are publicly available from the China Health and Retirement Longitudinal Study (CHARLS) project and can be downloaded after registering from: https://charls.charlsdata.com/pages/data/111/zh-cn.html.

## Abbreviations

ADL: Activities of daily living
CHARLS: China Health and Retirement Longitudinal Study
BADL: Basic activities of daily living
IADL: Instrumental activities of daily living
CESD-10: 10-Item Centre for Epidemiological Studies Depression Scale
SD: Standard deviation
CFI: Comparative Fit Index
TLI: Tucker Lewis Index
RMSEA: Root Mean Square Error of Approximation
SRMR: Standardized Root Mean-square Residual
