# Supplementary material for: Exploring the reciprocal relationship between activities of daily living disability and depressive symptoms among middle-aged and older Chinese people: a four-wave, cross-lagged model
Source: BMC Public Health. 2023 Jun 20;23:1180. doi: 10.1186/s12889-023-16100-0 (PMC10280867; doi:10.1186/s12889-023-16100-0)
Supplement: Supplementary file 1 — Additional file 1: Additional Fig. 1. An estimated autoregressive cross-lagged model of ADL disability and depressive symptoms over four waves between 2011 and 2018 in people with baseline age <60. Additional Fig. 2. An estimated autoregressive cross-lagged model of ADL disability and depressive symptoms over four waves between 2011 and 2018 in people with baseline age ≥60. Additional Tab. 1. Baseline characteristics of 4,124 study participants included in final analyses and 7,858 excluded due to data missing in the CHARLS. [file 12889_2023_16100_MOESM1_ESM.docx]

**Additional Figure**

**Additional Fig.1** An estimated autoregressive cross-lagged model of ADL disability and depressive symptoms over four waves between 2011 and 2018 in people with baseline age <60

ADL disability

Wave 1 (2011 year)

ADL disability

Wave 3 (2015 year)

ADL disability

Wave 2 (2013 year)

ADL disability

Wave 4 (2018 year)

Depressive symptoms

Wave 2 (2013 year)

Depressive symptoms

Wave 4 (2018 year)

Depressive symptoms

Wave 1 (2011 year)

Depressive symptoms

Wave 3 (2015 year)

p1 0.081***

q1 0.095***

o1 0.235***

o2 0.182***

o3 0.259***

o4 0.231***

p2 0.076***

q3 0.084***

q2 0.093***

p3 0.051***

Dotted lines with one head represent auto-regressive paths; Solid lines with one head represent cross-lag paths; Solid lines with two heads represent concurrent associations among variables at the same wave. To simplify the presentation, this model only presents cross-lag paths (paths p1, p2, p3 and q1, q2, q3) and concurrent paths between variables at the same survey (paths o1, o2, o3 and o4). Covariates included age, gender, locality, education, hours of sleep at night, marital status, nap after lunch, tobacco use, alcohol use, chronic disease, and social activity. Continuous variable: ADL disability and depressive symptoms. The path coefficients shown are standardized. ***P < 0.001

ADL: activities of daily living

**Additional Fig.2** An estimated autoregressive cross-lagged model of ADL disability and depressive symptoms over four waves between 2011 and 2018 in people with baseline age ≥60

ADL disability

Wave 1 (2011 year)

ADL disability

Wave 3 (2015 year)

ADL disability

Wave 2 (2013 year)

ADL disability

Wave 4 (2018 year)

Depressive symptoms

Wave 2 (2013 year)

Depressive symptoms

Wave 4 (2018 year)

Depressive symptoms

Wave 1 (2011 year)

Depressive symptoms

Wave 3 (2015 year)

s1 0.048***

t1 0.118***

r1 0.306***

r2 0.254***

r3 0.256***

r4 0.240***

s2 0.063***

t2 0.105***

s3 0.053***

t3 0.063***

Dotted lines with one head represent auto-regressive paths; Solid lines with one head represent cross-lag paths; Solid lines with two heads represent concurrent associations among variables at the same wave. To simplify the presentation, this model only presents cross-lag paths (paths s1, s2, s3 and t1, t2, t3) and concurrent paths between variables at the same survey (paths r1, r2, r3 and r4). Covariates included age, gender, locality, education, hours of sleep at night, marital status, nap after lunch, tobacco use, alcohol use, chronic disease, and social activity. Continuous variable: ADL disability and depressive symptoms. The path coefficients shown are standardized. ***P < 0.001

ADL: activities of daily living

**Additional Table**

**Additional Tab.1** Baseline characteristics of 4,124 study participants included in final analyses and 7,858 excluded due to data missing in the CHARLS

| Characteristic | Participants  Included | Participants  excluded | *P* value |
| --- | --- | --- | --- |
| Age, years (mean ± SD) | 57.57 ± 7.89 | 58.59 ± 9.75 | <0.001 |
| Gender (%) |  |  | <0.001 |
| Male | 2,038 (49.42) | 3,506 (44.64) |  |
| Female | 2,086 (50.58) | 4,348 (55.36) |  |
| Locality (%) |  |  | 0.079 |
| Rural | 2,640 (64.02) | 5,157 (58.98) |  |
| Urban | 1,484 (35.98) | 2,701 (41.02) |  |
| Education (%) |  |  | <0.001 |
| Not finish primary school and lower | 1,629 (39.50) | 3,861 (49.26) |  |
| Graduate from elementary school | 1,014(24.59) | 1,639 (20.91) |  |
| Graduate from middle school | 937 (22.72) | 1,563(19.94) |  |
| High school education and above | 544(13.19) | 775 (9.89) |  |
| Marital status (%) |  |  | <0.001 |
| Have a spouse | 3,778 (91.61) | 7,002 (89.21) |  |
| Not have a spouse | 346(8.39) | 847(10.79) |  |
| Hours of actual sleep at night (%) |  |  | 0.002 |
| <6h | 1,130(27.40) | 2,133(30.16) |  |
| 6h– (include 6h) | 872(21.14) | 1,487(21.03) |  |
| 7h– (include 7h) | 1,793(43.48) | 2,845(40.23) |  |
| >8h | 329(7.98) | 607(8.58) |  |
| Nap after lunch (%) |  |  | 0.086 |
| Yes | 2,097(50.85) | 3,753(52.53) |  |
| No | 2,027(49.15) | 3,392(47.47) |  |
| Tobacco use (%) |  |  | 0.544 |
| Yes | 1,356(32.88) | 2,610(33.43) |  |
| No | 2,768(67.12) | 5,197(66.57) |  |
| Alcohol use (%) |  |  | 0.226 |
| Yes | 1,625(39.40) | 2,985(38.27) |  |
| No | 2,499(60.60) | 4,815(61.73) |  |
| Chronic disease (%) |  |  | 0.296 |
| Yes | 2,809(68.11) | 5,172(67.17) |  |
| No | 1,315(31.89) | 2,528(32.83) |  |
| Social activity (%) |  |  | <0.001 |
| Yes | 2,237(54.24) | 3,540(49.43) |  |
| No | 1,887(45.76) | 3,621(50.57) |  |
